# Chemical Diversity in Basil (*Ocimum* sp.) Germplasm

**DOI:** 10.1155/2015/352638

**Published:** 2015-01-01

**Authors:** Andréa Santos da Costa, Maria de Fátima Arrigoni-Blank, José Luiz Sandes de Carvalho Filho, Aléa Dayane Dantas de Santana, Darlisson de Alexandria Santos, Péricles Barreto Alves, Arie Fitzgerald Blank

**Affiliations:** ^1^Department of Agronomic Engineering, Federal University of Sergipe, Avenida Marechal Rondon s/n, 49100-000 São Cristóvão, SE, Brazil; ^2^Department of Agronomy, Federal Rural University of Pernambuco, Campus Universitário, 52171-900 Recife, PE, Brazil; ^3^Department of Chemistry, Federal University of Sergipe, Avenida Marechal Rondon s/n, 49100-000 São Cristóvão, SE, Brazil

## Abstract

The present study aimed to chemically characterize 31 accessions and seven cultivars of basil. The percentage composition of the essential oils of the accessions and cultivars was based on the 14 most abundant constituents: 1,8-cineole, linalool, methyl chavicol, neral, nerol, geraniol, geranial, methyl cinnamate, *β*-bourbonene, methyl eugenol, *α*-*trans*-bergamotene, germacrene-D, *epi*-*α*-cadinol, and *δ*-cadinene. The genetic materials were classified into eight clusters according to the chemical composition of the essential oils: Cluster 1—mostly linalool and 1,8-cineole; Cluster 2—mostly linalool, geraniol, and *α*-*trans*-bergamotene; Cluster 3—mostly linalool, methyl chavicol, methyl cinnamate, and *β*-bourbonene; Cluster 4—mostly linalool, methyl chavicol, *epi*-*α*-cadinol, and *α*-*trans*-bergamotene; Cluster 5—mainly linalool, methyl eugenol, *α*-*trans*-bergamotene, and *epi*-*α*-cadinol; Cluster 6—mainly linalool, geraniol, and *epi*-*α*-cadinol; Cluster 7—mostly linalool and methyl chavicol; Cluster 8—mainly geranial and neral.

## 1. Introduction

The genus* Ocimum* L. (Lamiaceae) comprises more than 30 species that are found in tropical and subtropical regions [1]. Due to their economic importance, the most cultivated species in the world are* O. x citriodorum* Vis.,* O. americanum* L.,* O. basilicum* L.,* O. gratissimum* L.,* O. minimo* L., and* O. tenuiflorum* L. [2].

Basil (*Ocimum basilicum* L.) is a medicinal plant traditionally used for the treatment of respiratory and intestinal problems and kidney malfunction [3]. Basil is economically important due to the use of its essential oil in hygiene and cleaning products, perfumes, and cosmetics and as a local anesthetic and antiseptic [4]. Furthermore, basil essential oil has been tested in the control of plant pests [5, 6] and diseases [7] and has been shown to act as an antioxidant [8] and an antimicrobial [9].

Basil has a complicated taxonomy due to the numerous varieties of cultivars within the species that do not differ significantly in morphology. Thus, the classification of genotypes only by morphological features becomes difficult due to anthropogenic interference with selection, cultivation, and hybridization [10]. Due to the hybridization of several species and varieties, there is a wide variability of the chemical constituents [11]. Chemical characterization can be used to separate the accessions based on the presence or concentration of specific substances and to determine the intrinsic variability or variability among accessions of the same species [10]. Despite the wide variation in the chemical composition of basil essential oil within the same species, monoterpenes and phenylpropanoids predominate [12, 13].

Genotype characterization based on the chemical constitution of the essential oil has been used in several cultures such as* Zingiber officinale* [14] and* Hyptis suaveolens* [15], including plants of the genus* Ocimum*, with emphasis on* O. basilicum* [16]. Several studies assessing the chemical composition of 18 basil essential oils observed that the samples distributed into seven distinct types, each one presenting as the major volatile compound among the following: linalool, methyl cinnamate, methyl cinnamate/linalool, methyl eugenol, citral, methyl chavicol (estragole), and methyl chavicol/citral.

A total of 27 basil cultivars were characterized according to the chemical composition of their essential oils, and the cultivars were grouped into five different types: eugenol/linalool (30–35% linalool and 12–20% eugenol); linalool (52–66%); estragole/linalool (22–38% estragole and 21–37% linalool);* (Z)* methyl cinnamate (19–38%); and estragole (38–95%) [4].

The chemical characterization of 38 basil genotypes resulted in seven groups: linalool (19–73%); linalool/eugenol (28–66% linalool and 5–29% eugenol); methyl chavicol (20–72% methyl chavicol); methyl chavicol/linalool (8–29% methyl chavicol and 8–53% linalool); methyl eugenol/linalool (two accessions with 37% and 91% methyl eugenol and 60% and 15% linalool); methyl cinnamate/linalool (9.7% methyl cinnamate and 31% linalool); and bergamotene (one accession with bergamotene as the major constituent) [17].

Morphological and agronomic characterization of 55 basil accessions showed genetic variability in* Ocimum* sp. The present study observed genotypic variations in relation to the content and yield of essential oil, and promising genotypes were noted for the development of cultivars with high content and yield of essential oil rich in linalool and other active ingredients [18].

These comparative studies show a high variation in the chemical composition of basil essential oil and how important it is to conduct research investigating these chemical characterizations because the identification of types of basil helps in pharmacological, medicinal, and phytopathological studies. Thus, the present study aimed to chemically characterize the volatile constituents of accessions and cultivars of* O. basilicum* L. grown in Northeastern Brazil.

## 2. Materials and Methods

### 2.1. Plant Material and Growing Conditions

The experiment was conducted at the Experimental Farm “Rural Campus of the UFS.” A randomized complete block design with two repetitions was used to evaluate 38 genotypes of* O. basilicum* with 31 accessions provided by the North Central Regional PI Station, Iowa State University, USA, and seven commercial cultivars donated by the companies Topseed and Johnny's Selected Seeds (Table 1). Each replication consisted of a 2.5 m row with 0.5 m space between plants and 0.8 m between rows.

The seedlings were grown in a greenhouse with a 50% shading screen in polystyrene trays with 128 alveoli. The substrate mixture used was composed of coconut powder, cattle manure, and carbonized rice husk at a 1 : 1 : 1 ratio, which had been chosen during preliminary tests of the substrates' composition. Seven days after sowing, 90% of the accessions had emerged, and 30 days after sowing, the seedlings were transplanted to the field. For soil preparation, cleaning and subsequent liming with dolomitic limestone were performed to reach 60% base saturation two months before the establishment of the experiment. Poultry manure was used as a nutrient source at a ratio of 27.5 m^3^ h^−1^.

Three months after cultivation, the plants were harvested, and the leaves were placed to dry in an oven with air circulation at a temperature of 40°C for five days.

### 2.2. Distillation and Chemical Analysis of Essential Oils

The extraction of the essential oil from dried leaves was performed by the hydrodistillation method with a Clevenger-type apparatus [19] for 160 minutes [20]. A total of 50 g dry leaves were used for each flask.

Oil sample analysis was performed on a Shimadzu QP5050A (Shimadzu Corporation, Kyoto, Japan) system comprising a AOC-20i autosampler and gas chromatograph interfaced to a mass spectrometer (GC-MS) instrument employing the following conditions: column J&W Scientific DB-5MS (Folsom, CA, USA) fused silica capillary column (30 m × 0.25 mm i.d. × 0.25 *μ*m film, composed of 5% phenylmethylpolysiloxane), using helium carrier gas with a flow of 1.2 mL·min^−1^. The temperature was programmed to be maintained at 50°C for 1.5 min, followed by an increase of 4°C/min up to 200°C and then at 15°C/min up to 250°C, keeping this temperature constant for 5 min. The injector temperature was 250°C, and the detector temperature was 280°C; a volume of 0.5 *μ*L ethyl acetate was injected; the partition rate of the injected volume was 1 : 87 at 64.20 kPa column pressure. The mass spectrometry conditions were quadrupole ion detector operating by electron impact at an impact energy of 70 eV; sweep speed of 1.000; sweep interval of 0.85 fragments/s; and fragments detected in the range 40–550 Da.

The quantitative analysis of the chemical components was performed by flame ionization detector (FID) gas chromatography using a Shimadzu GC-17A (Shimadzu Corporation, Kyoto, Japan) under the following operating conditions: ZB-5MS capillary column (5% phenyl-arylene-95%-dimethylpolysiloxane) of fused silica (30 m × 0.25 mm i.d. × 0.25 *μ*m film) from Phenomenex (Torrance, CA, USA) under the same conditions as described for GC-MS. The quantification of each component was calculated by area normalization. Compounds concentrations were calculated from the GC relative peak areas (%) and they were arranged in order of GC elution.

The essential oil components were identified by comparing their mass spectra with spectra available in the database of the equipment (NIST05, NIST21, and WILEY8). These libraries allowed comparisons of the spectral data, which had a similarity index of 80%. Furthermore, the measured retention indices were compared to the indices in the literature [21]. The relative retention indices (RRIs) are determined using equation [22] relative to a homologous series of *n*-alkanes (C9–C18), injected under the chromatographic conditions described above.

### 2.3. Statistical Analysis

The chemical composition data were analyzed by two multivariate analysis methods: principal component analysis (PCA) and arrangements analysis (cluster) based on the similarity between individuals and their constituent distribution, using the Statistica software version 7.0 Statsoft company.

## 3. Results and Discussion

The 14 principle compounds were identified from the essential oils of 31 accessions and seven cultivars and are listed according to elution order in Table 2. Linalool was the main constituent of the essential oil of most accessions and cultivars of basil. In the cultivars “Genovese,” “Fino Verde,” “Red Rubin,” and “Osmin Purple” and in the accessions NSL6421, PI 197442, PI 358464, PI 368698, and PI 414194, the linalool concentration was over 60% (Table 2). Linalool was also the major compound in basil essential oil in Italy [23, 24] and in the United States [17].

The cultivar “Cinnamon” stood out for its high concentration of methyl cinnamate, 31%, a chemical constituent with a cinnamon aroma. This constituent was present in only three accessions and always at levels below 6% (Table 2). This result is similar to the result reported for* Ocimum americanum* var.* americanum* in Brazil [25],* O. canum* [26], and “Cinnamon” in Australia [27].

The accessions PI 414196, PI 176 646, PI 211586, PI 414193, and PI 414198 showed epi-*α*-cadinol levels above 30%; accession PI 358466 presented 46% geraniol, and accession PI 172996 presented 45% methyl chavicol (Table 2). The presence of methyl chavicol in basil essential oil has been reported in genotypes in Yemen, USA, Thailand, United Kingdom [28], Brazil [25], and local varieties in Turkey [16].

The variety “Sweet Dani” contained high levels of neral (33.98%) and geranial (43%) constituents (Table 2), confirming what had already been reported [29] on the high concentrations of citral (68%) in this cultivar. In a study genetically characterizing* Ocimum species* [30], the cultivars that had citral as the major compound were grouped in the* Ocimum x citriodorum* Vis. cluster; thus, the cultivar “Sweet Dani” is a natural hybrid between* O. basilicum* and* O. Americanum.* Because all of the accessions and cultivars in the present study were grown in the same environment, variations in the concentrations of the compounds among the accessions and cultivars may have been a consequence of the place of origin; chemical variations may occur among accessions and cultivars of basil from different regions [31, 32].

**Figure 1:**
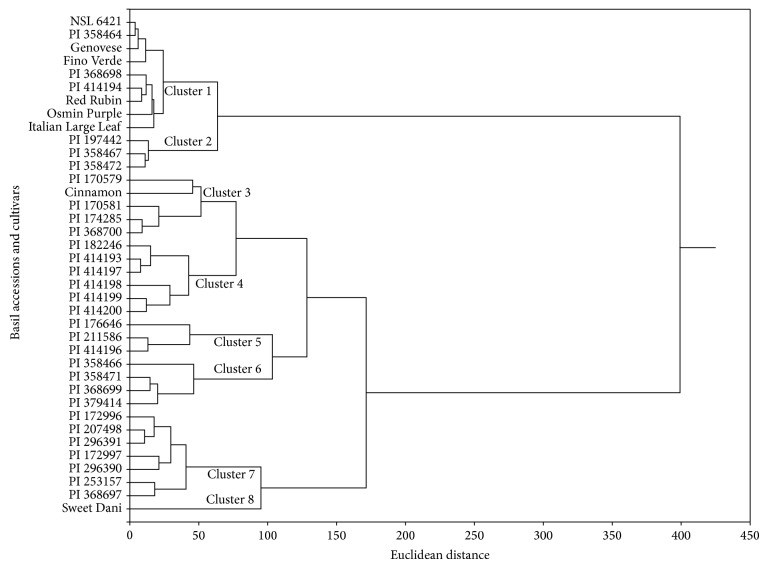
Bidimensional dendrogram representing the similarity between 31 accessions and seven cultivars of* Ocimum basilicum* for the chemical composition of the essential oils.

According to our chemical analyses (Table 2), the compounds found in greatest quantity among the accessions were 1,8-cineole, linalool, methyl chavicol, neral, nerol, geraniol, geranial, methyl cinnamate, *β*-bourbonene, methyl eugenol, *α*-*trans*-bergamotene, germacrene-D, epi-*α*-cadinol, and *δ*-cadinene, which defined the formation of eight clusters according to chemical composition and differentiation by cluster analysis (Figure 1).

**Figure 2:**
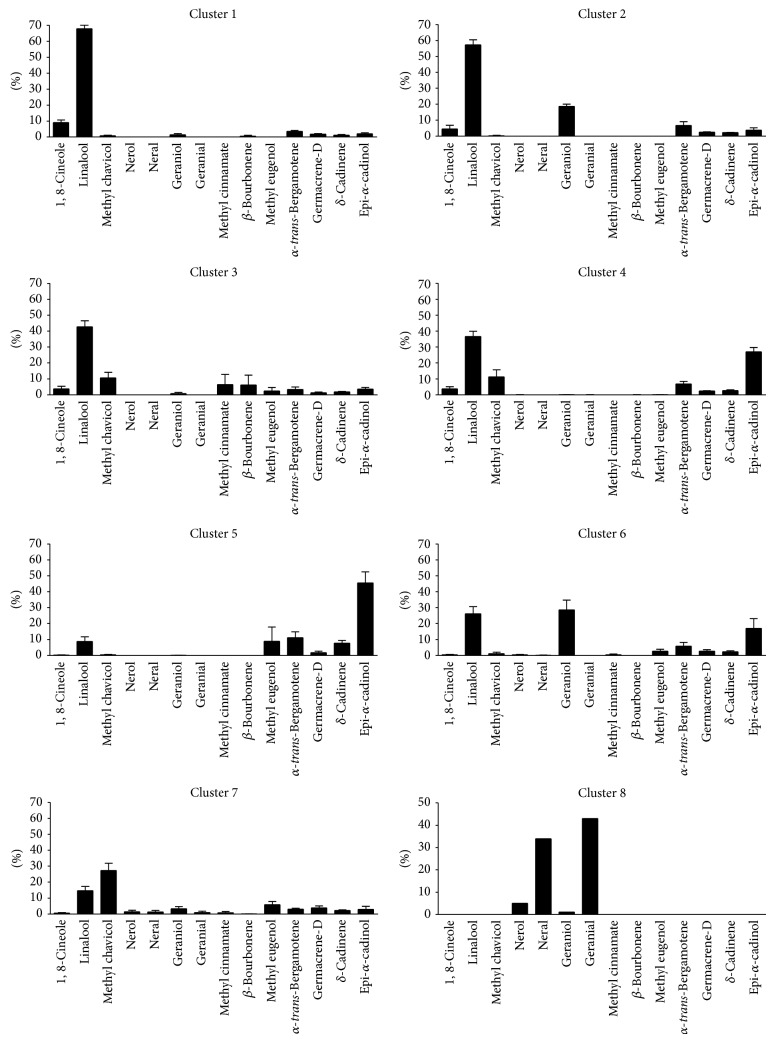
Means of the 14 most important chemical constituents of the essential oils of clusters 1 to 8 of* Ocimum basilicum* (vertical lines show ± SEM).

Considering the similarities of the chemical constituents of the essential oils of 31 accessions and seven cultivars of basil, the clusters were characterized as follows: Cluster 1 NSL-6421, PI-358 464, “Genovese,” “Fino Verde,” PI-368698, PI-414194, “Red Rubin,” “Osmin Purple,” and “Italian Large Leaf” with linalool and 1,8-cineole as the major compounds; Cluster 2: PI-197442, PI-358472, and PI-358467 with linalool, geraniol, and *α*-*trans*-bergamotene as the major compounds; Cluster 3: PI-170579, “Cinnamon,” PI-170581, PI-174285, and PI-368700 with linalool, methyl chavicol, methyl cinnamate, and *β*-bourbonene as the major compounds; Cluster 4: PI-182246, PI-414193, PI-414197, PI-414198, PI-414199, and PI-414200 with linalool, methyl chavicol,* epi*-*α*-cadinol, and *α*-*trans*-bergamotene as the major compounds; Cluster 5: PI-176646, PI-211586, and PI-414196 with linalool, methyl eugenol, *α*-*trans*-bergamotene, and* epi*-*α*-cadinol as the major compounds; Cluster 6: PI-358466, PI-358471, PI-368699, and PI-379414 with linalool, geraniol, and* epi*-*α*-cadinol as the major compounds; Cluster 7: PI-172996, PI-207498, PI-296391, PI-172997, PI-296390, PI-253157, and PI-368697 with linalool and methyl chavicol as the major compounds; and Cluster 8: “Sweet Dani” with geranial and neral as the major compounds (Figure 2). This last cluster showed similar results to the study of [29] wherein citral (geranial and neral) was the major compound of the genotype* O. x citriodorum* Vis.

The grouping of cultivars and accessions of basil through the chemical composition of the essential oil provides the market with new groups of plants with similar major active compounds, thus broadening the sources of raw materials for the industries. Various activities of* O. basilicum* essential oil are being investigated, such as its antibacterial effects against* Escherichia coli* with oil from the cultivar Maria Bonita (PI-197442-S3-bulk 5) [33]. Thus, it can be inferred that Cluster 2 accessions would also express this activity.

**Figure 3:**
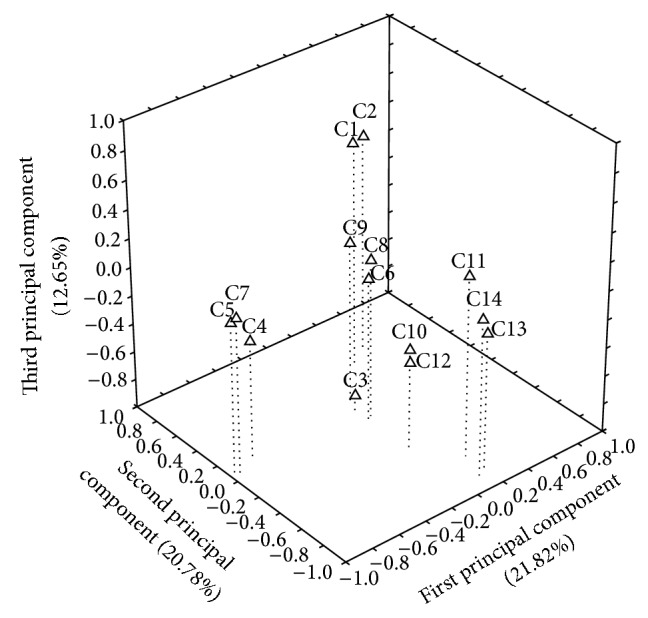
Distribution of the chemical constituents of the essential oil of* Ocimum basilicum* in relation to the three principal components through analysis of the principal component analysis (PCA) (C1 = 1,8-cineole, C2 = linalool, C3 = methyl chavicol, C4 = nerol, C5 = neral, C6 = geraniol, C7 = geranial, C8 = methyl cinnamate, C9 = β-bourbonene, C10 = methyl eugenol, C11 = α-*trans*-bergamotene, C12 = germacrene-D, C13 = δ-cadinene, and C14 =* epi*-α-cadinol).

According to principal component analysis (Figure 3), the first principal component accounted for 21.82% of the total variance and was positively related to *δ*-cadinene (*r* = 0.80) and epi-*α*-cadinol (*r* = 0.85) and negatively related to germacrene-D (*r* = −0.10) and methyl eugenol (*r* = −0.20). The second principal component represented 20.87% of the total variance and was positively related to geranial (*r* = 0.98), (*r* = 0.99) and nerol (*r* = 0.80). The third principal component accounted for 12.65% of the total variance and was positively related to linalool (*r* = 0.80) and 1,8-cineole (*r* = 0.70) and negatively related to methyl chavicol (*r* = −0.90), geraniol (*r* = −0.20), and methyl cinnamate (*r* = −0.10).

We observed a correlation greater than 0.50 among some of the chemical constituents of basil essential oil in the accessions and cultivars under study (Table 3). The correlation between geranial and neral was positive and very strong (0.99), suggesting that these constituents that are present in the oil of the cultivar “Sweet Dani” are closely correlated, indicating that, possibly, when selecting this cultivar aimed at producing an oil with a higher content of geranial, neral will also be selected.

Nerol and neral as well as nerol and geranial, all present in the cultivar “Sweet Dani,” showed a correlation coefficient of 0.67 and 0.57 (moderate), respectively, for each pair. Epi-*α*-cadinol showed strong positive correlation with *δ*-cadinene (0.80) among Cluster 5 accessions (PI-176646, PI-211586, and PI-414196). A moderate positive correlation was observed between 1,8-cineole and linalool (0.66) among the accessions and cultivars of Cluster 1 (NSL-6421, PI-358464, PI-368698, PI-414194, PI-197442, PI-358472, PI-358467, “Red Rubin,” “Osmin Purple,” “Italian Large Leaf,” “Genovese,” and “Fino Verde”), and these components were the major compounds in Cluster 1.

Most of the constituents showed correlation coefficients that were classified as weak or very weak, which indicates a low correlation among the variables under study.

## 4. Conclusions

The accessions and cultivars were classified according to the chemical composition of their essential oils, forming eight clusters: Cluster 1—mostly linalool and 1,8-cineole; Cluster 2—mostly linalool, geraniol, and *α*-*trans*-bergamotene; Cluster 3—mostly linalool, methyl chavicol, methyl cinnamate, and *β*-bourbonene; Cluster 4—mostly linalool, methyl chavicol,* epi*-*α-*cadinol, and *α*-*trans*-bergamotene; Cluster 5—mainly linalool, methyl eugenol, *α*-*trans*-bergamotene, and* epi*-*α*-cadinol; Cluster 6—mainly linalool, geraniol, and* epi*-*α*-cadinol; Cluster 7—mostly linalool and methyl chavicol; Cluster 8: mainly geranial and neral. We observed that compounds such as linalool, geraniol, methyl cinnamate, geranial, neral, methyl chavicol, and* epi*-*α*-cadinol showed concentrations above 30% in some of the accessions and cultivars.

## Figures and Tables

**Table 1:** Identification and origin of analysed basil accessions and cultivars.

Accession	Species	Origin
NSL 6421	*Ocimum basilicum *	North Central Regional PI Station
PI 170579	*Ocimum basilicum *	North Central Regional PI Station
PI 170581	*Ocimum basilicum *	North Central Regional PI Station
PI 172996	*Ocimum basilicum *	North Central Regional PI Station
PI 172997	*Ocimum basilicum *	North Central Regional PI Station
PI 174285	*Ocimum basilicum *	North Central Regional PI Station
PI 176646	*Ocimum basilicum *	North Central Regional PI Station
PI 182246	*Ocimum basilicum *	North Central Regional PI Station
PI 197442	*Ocimum basilicum *	North Central Regional PI Station
PI 207498	*Ocimum basilicum *	North Central Regional PI Station
PI 211586	*Ocimum basilicum *	North Central Regional PI Station
PI 253157	*Ocimum basilicum *	North Central Regional PI Station
PI 296390	*Ocimum basilicum *	North Central Regional PI Station
PI 296391	*Ocimum basilicum *	North Central Regional PI Station
PI 358464	*Ocimum basilicum *	North Central Regional PI Station
PI 358466	*Ocimum basilicum *	North Central Regional PI Station
PI 358467	*Ocimum basilicum *	North Central Regional PI Station
PI 358471	*Ocimum basilicum *	North Central Regional PI Station
PI 358472	*Ocimum basilicum *	North Central Regional PI Station
PI 368697	*Ocimum basilicum *	North Central Regional PI Station
PI 368698	*Ocimum basilicum *	North Central Regional PI Station
PI 368699	*Ocimum basilicum *	North Central Regional PI Station
PI 368700	*Ocimum basilicum *	North Central Regional PI Station
PI 379414	*Ocimum basilicum *	North Central Regional PI Station
PI 414193	*Ocimum basilicum *	North Central Regional PI Station
PI 414194	*Ocimum basilicum *	North Central Regional PI Station
PI 414196	*Ocimum basilicum *	North Central Regional PI Station
PI 414197	*Ocimum basilicum *	North Central Regional PI Station
PI 414198	*Ocimum basilicum *	North Central Regional PI Station
PI 414199	*Ocimum basilicum *	North Central Regional PI Station
PI 414200	*Ocimum basilicum *	North Central Regional PI Station
“Genovese”	*Ocimum basilicum *	Topseed (Brazil)
“Sweet Dani”	*Ocimum x citriodorum* ^*^	Johnny's Selected Seeds Co
“Cinnamon”	*Ocimum basilicum *	Johnny's Selected Seeds Co.
“Fino Verde”	*Ocimum basilicum *	Johnny's Selected Seeds Co.
“Red Rubin”	*Ocimum basilicum *	Johnny's Selected Seeds Co.
“Osmin Purple”	*Ocimum basilicum *	Johnny's Selected Seeds Co.
“Italian Large Leaf”	*Ocimum basilicum *	Johnny's Selected Seeds Co.

^*^According to [30].

**Table 2:** Chemical composition of the essential oil of 31 accessions and seven cultivars of basil.

Genotype	Chemical compounds (%)													
	C1	C2	C3	C3	C5	C6	C7	C8	C9	C10	C11	C12	C13	C14
	RRI													
	1033	1098	1195	1228	1240	1255	1270	1379	1384	1401	1436	1480	1513	1640
NSL 6421	8.76	72.15	3.00	0.00	0.00	0.51	0.00	0.00	0.00	0.00	2.37	1.12	1.02	0.00
PI 170579	8.11	38.62	0.57	0.00	0.00	0.00	0.00	0.00	30.75	0.00	0.00	0.62	1.15	5.17
PI 170581	2.46	38.86	13.37	0.00	0.00	3.23	0.00	0.00	0.00	11.19	3.68	0.00	2.06	4.81
PI 172996	0.00	7.28	45.82	0.00	0.00	1.79	0.00	0.00	0.00	2.72	3.79	3.58	2.73	0.00
PI 172997	0.00	9.11	28.76	0.00	0.00	0.15	0.00	0.00	0.15	8.38	0.00	10.48	2.45	14.17
PI 174285	0.34	53.44	19.47	0.00	0.00	0.00	0.00	0.00	0.15	0.00	3.58	2.62	2.45	5.65
PI 176646	0.34	12.17	0.70	0.00	0.00	0.15	0.00	0.00	0.00	26.84	15.91	0.00	4.53	31.76
PI 182246	0.34	34.15	0.70	0.00	0.00	0.15	0.00	0.00	0.00	0.10	9.91	2.62	2.45	27.35
PI 197442	8.00	61.57	0.70	0.00	0.00	19.40	0.00	0.00	0.00	0.00	2.09	2.62	2.15	1.10
PI 207498	1.58	13.64	35.88	0.00	0.00	0.74	0.00	0.00	0.00	4.44	3.75	3.93	2.09	0.00
PI 211586	0.00	11.27	0.00	0.00	0.00	0.00	0.00	0.00	0.00	0.00	13.52	2.62	7.99	51.22
PI 253157	0.00	19.67	21.36	6.88	4.53	6.30	0.00	0.65	0.21	1.85	3.75	2.05	0.99	0.00
PI 296390	2.32	10.24	19.68	0.00	0.00	0.67	0.00	0.00	0.00	16.85	3.57	2.13	3.46	0.00
PI 296391	0.00	16.54	30.78	2.71	4.87	2.63	6.30	0.00	0.00	3.55	3.03	2.74	1.68	1.29
PI 358464	10.17	70.94	0.00	0.00	0.00	0.00	0.00	0.00	0.00	0.00	0.92	1.26	0.69	0.00
PI 358466	0.00	17.30	3.79	1.33	0.48	46.27	0.00	1.86	0.00	1.45	4.05	1.69	1.18	0.00
PI 358467	5.46	50.97	0.00	0.00	0.00	16.00	0.00	0.00	0.00	0.00	7.91	2.20	2.21	5.15
PI 358471	1.31	24.58	0.00	0.00	0.00	23.46	0.00	0.00	0.00	4.29	3.27	0.85	3.56	28.41
PI 358472	0.00	59.17	0.00	0.00	0.00	20.60	0.00	0.00	0.00	0.00	9.91	2.62	2.45	5.22
PI 368697	0.00	26.36	9.10	0.00	0.00	10.54	0.00	5.20	0.00	3.29	3.74	2.49	2.52	4.60
PI 368698	14.19	68.65	0.00	0.00	0.00	7.57	0.00	0.00	0.00	0.00	3.75	2.62	0.00	0.00
PI 368699	0.00	24.64	0.00	0.00	0.00	17.84	0.00	0.00	0.00	5.10	13.08	5.45	2.40	20.53
PI 368700	0.95	49.26	15.05	0.00	0.00	0.91	0.00	0.66	0.00	0.94	0.94	1.73	2.05	0.00
PI 379414	0.34	38.22	0.70	0.24	0.00	26.78	0.00	0.00	0.00	0.00	2.90	2.62	2.45	19.38
PI 414193	5.83	44.22	0.00	0.00	0.00	0.00	0.00	0.00	0.00	0.00	9.41	2.62	5.18	32.66
PI 414194	6.10	65.62	0.00	0.00	0.00	0.00	0.00	0.00	2.36	0.00	7.91	2.62	2.45	3.98
PI 414196	0.34	2.93	0.70	0.00	0.00	0.00	0.00	0.00	0.00	0.00	4.06	2.62	10.68	53.60
PI 414197	7.10	44.13	5.44	0.00	0.00	0.15	0.09	0.00	0.00	0.10	5.95	2.52	2.35	29.33
PI 414198	0.34	22.46	26.33	0.00	0.00	0.15	0.00	0.00	0.00	0.00	2.36	2.62	2.55	34.59
PI 414199	6.66	35.98	20.70	0.00	0.00	0.00	0.00	0.00	0.00	0.00	10.91	1.62	1.45	19.83
PI 414200	1.83	38.68	14.40	0.24	0.00	0.15	0.09	0.00	0.15	0.10	2.68	2.62	2.35	17.94
Genovese	11.85	75.09	0.00	0.00	0.00	0.00	0.00	0.00	0.00	0.00	3.31	0.45	0.65	1.68
Sweet Dani	0.00	0.00	0.00	5.04	33.98	1.06	43.02	0.00	0.00	0.00	0.00	0.00	0.00	0.00
Cinnamon	7.00	33.67	4.70	0.00	0.00	0.00	0.00	31.64	0.00	0.00	8.91	1.58	1.87	2.29
Fino Verde	3.30	74.84	0.00	0.00	0.00	3.45	0.00	0.00	0.00	0.00	3.20	2.62	2.25	3.50
Red Rubin	13.15	65.60	0.36	0.00	0.00	0.00	0.00	0.00	0.00	0.00	5.19	0.94	0.57	1.79
Osmin Purple	0.00	63.51	0.56	0.00	0.00	0.85	0.00	0.00	3.13	0.00	0.53	3.95	0.91	2.41
Italian Large Leaf	13.85	54.44	2.70	0.00	0.00	0.00	0.00	0.00	0.00	0.00	4.26	1.13	2.37	4.86

RRI: Relative Retention Index. Identification of the chemical constituents: C1 = 1,8-cineole, C2 = linalool, C3 = methyl chavicol, C4 = nerol, C5 = neral, C6 = geraniol, C7 = geranial, C8 = methyl cinnamate, C9 = *β*-bourbonene, C10 = methyl eugenol, C11 = *α*-*trans*-bergamotene, C12 = germacrene-D, C13 = *δ*-cadinene, and C14 = *epi*-*α*-cadinol.

**Table 3:** Correlation coefficients for the most abundant chemical constituents in basil essential oil.

Chemical compound	C2	C3	C4	C5	C6	C7	C8	C9	C10	C11	C12	C13	C14
C1	0.66	−0.36	−0.26	−0.18	−0.22	−0.16	0.08	0.15	−0.28	−0.08	−0.32	−0.34	−0.28
C2		−0.46	−0.34	−0.32	−0.05	−0.30	−0.06	0.04	−0.42	−0.13	−0.21	−0.43	−0.38
C3			0.15	−0.05	−0.24	−0.07	−0.05	−0.13	0.18	−0.24	0.38	−0.07	−0.18
C4				0.67	0.06	0.57	−0.04	−0.06	−0.05	−0.19	−0.14	−0.25	−0.22
C5					−0.07	0.99	−0.04	−0.04	−0.07	−0.22	−0.21	−0.23	−0.16
C6						−0.08	−0.04	−0.11	−0.06	0.03	0.01	−0.10	−0.10
C7							−0.04	−0.04	−0.07	−0.22	−0.21	−0.21	−0.14
C8								−0.04	−0.07	0.15	−0.07	−0.05	−0.13
C9									−0.09	−0.22	−0.14	−0.12	−0.09
C10										0.28	−0.01	0.17	0.08
C11											−0.06	0.38	0.48
C12												0.12	0.10
C13													0.80
C14													1.00

Identification of the chemical constituents: C1 = 1,8-cineole, C2 = linalool, C3 = methyl chavicol, C4 = nerol, C5 = neral, C6 = geraniol, C7 = geranial, C8 = methyl cinnamate, C9 = *β*-bourbonene, C10 = methyl eugenol, C11 = *α*-*trans*-bergamotene, C12 = germacrene-D, C13 = *δ*-cadinene, and C14 = *epi*-*α*-cadinol.
